# Distance to Health Care Facilities, Lifestyle Risk Factors, and Stage at Diagnosis in relation to Geographic Pattern of Esophageal Cancer in Tanzania, 2006–2016

**DOI:** 10.1155/2022/7873588

**Published:** 2022-08-22

**Authors:** Richard Watkins, Ghada A. Soliman, Julius Mwaiselage, Crispin Kahesa, Khadija Msami, Mark L. Wilson

**Affiliations:** ^1^Department of Epidemiology, School of Public Health, University of Michigan, Ann Arbor, MI 48109, USA; ^2^Department of Environmental, Occupational and Geospatial Health Sciences, Graduate School of Public Health and Health Policy, City University of new York (CUNY), 55 W, 125th Street, New York, NY 10027, USA; ^3^Ocean Road, Ocean Road Cancer Institute (ORCI), Dar es Salaam, Tanzania

## Abstract

Esophageal cancer is an aggressive, often deadly disease globally that represents a significant health problem in Tanzania. The WHO reported 604,100 new esophageal cancer cases worldwide during 2020 and 544,076 deaths (Sung, 2021; World Health Organization, 2020). In Eastern Africa, 16,137 cases and 15,188 deaths were related to this disease in 2020. Esophageal cancer is associated with various etiologic risk factors, and access to the disease treatment is a major barrier to survival. This study examined associations between the prevalence of four geographically stratified, population-level, etiologic risk factors (tobacco use, unprotected water use, solid fuel source use, and poverty), as well as two access-to-care predictors (persons per hospital and distance from residence to where esophageal cancer treatment occurs). Regional- and coarser-scale zonal incidence rates were calculated for 2006 through 2016 and evaluated for geographic differences in relation to risk factors and access to care predictors using Poisson regression. Differences in the geographic distribution of esophageal cancer were observed. Distance from the region of residence to the treatment center (Ocean Road Cancer Institute) was statistically associated with the geographic pattern of esophageal cancer incidence. Further research into etiologic risk factors, dietary practices, and nutrition is needed to better understand the associations with esophageal cancer in Tanzania and other parts of Eastern Africa.

## 1. Introduction

Esophageal cancer (EC) represents an important health problem because it often presents at an advanced stage at diagnosis, and limited effective treatment options are available [1–3]. Globally, EC is the seventh most common cancer in men and the thirteenth most common in women [4]. In 2020, EC accounted for 3.1% of new cancer cases and 5.5% of cancer deaths, globally [4, 5]. In Eastern Africa, the 2020 age-standardized incidence of EC was 8.4 per 100,000 in men and 6.4 per 100,000 in women [4]. Cancer in Tanzania is an increasing health problem as part of the growing incidence of noncommunicable diseases due to the epidemiological transition [6]. Esophageal cancer, in particular, is a significant cancer burden in Tanzania, representing the third most common cancer in men and the fourth most common cancer in women [5]. Although the geographic distribution of places of residence of EC patients in Tanzania was previously reported in a small study [7], no investigation has been conducted to explore further possible links between places of residence and their regional risk factors.

Various etiologic factors and treatment access have been shown to influence the distribution and incidence of this disease [8–12]. EC is not regularly diagnosed at earlier stages in Tanzania and similar impoverished settings, because routine screening and medical examination of patients are often lacking. Patients have limited access to local medical care, especially in rural settings. They frequently neglect seeking treatment of early symptoms, and the limited-resource health facilities typically lack efficient diagnostic methods such as endoscopic examination. Gabel et al. categorized the administrative Regions in Tanzania based on the EC incidence rate and provided the presented demographic description of the study population and histopathologic type of EG as variation by gender and age group [7].

Esophageal cancer in Tanzania was previously studied using registry data during 2006–2016 from the Ocean Road Cancer Institute (ORCI), the source of data for the present study [7]. ORCI is the largest cancer hospital in Tanzania, located in the capital city of Dar es Salaam. In addition, an analysis of region-specific cases of EC from ORCI registry data from 2006 to 2013 showed differences in incidence among Regions [7]. Further exploration of the associations between the Region-specific risk factor prevalence and EC differences may help explain geographic patterns of EC in Tanzania [7]. In addition, addressing the potential for differential rates of diagnosis of EC based on access to care from regional hospitals should help to evaluate the possible underreporting of cases. Analysis of distances to ORCI may be predictive of the regional incidence rate of EC. Unfortunately, referrals to ORCI from distant areas are not as simple as we would have liked. As reported in our previous study from Tanzania [13], even when early detection tools are available at local clinics for other cancers, such as cervical cancer, and patients are given referral notes to ORCI, patients often do not use the referral notes. Most patients self-refer themselves after the severity of symptoms has increased. Therefore, the distance from their local place of residence to ORCI is the best possible measure for estimating differential referrals.

Therefore, we conducted this study using data on esophageal cancer cases from the ORCI registry, the only national cancer hospital in Tanzania. Patients were referred from different parts of the country for chemo- and radiation therapy. We used the dataset of all esophageal cancer patients seen at ORCI from 2006 through 2016 to explore whether the geographic residence of patients could be linked to specific regional risk factors. We also investigated whether the distance from the patient's residence was related to delayed treatment of advanced-stage EC patients.

## 2. Methods

ORCI patient registry data were combined with population-level data from the Tanzanian Demographic Health Survey and the Tanzanian Census, as they are essential to developing a comprehensive approach to understanding the epidemiology and management outcomes of esophageal cancer in Tanzania. We used data from the 2002 census as the population estimates were available for each region by year from 2006-2016. The more up-to-date 2012 census did not have population estimates by region per year. We decided that capturing the projected changes in the population by year when calculating the incidence rate was more important than using the more up-to-date census population from 2012 for all years between 2006 and 2016, as the relative change in population yearly would not bias the incidence rate as the population grew [14].

It is known that esophageal cancer differs by Region in Tanzania, which could be explained by greater risk exposure or improved diagnosis, leading to more esophageal cancer being recorded. According to the African Esophageal Cancer Consortium, several risk factors for esophageal cancers are identified in East Africa [15]. Therefore, comparing the risk factors in other African countries with high rates of EC to those in Tanzania will provide knowledge about the EC epidemic in Tanzania [16, 17].

### 2.1. Study Population and Setting

This study was conducted at the Ocean Road Cancer Institute (ORCI). The study included all patients with EC seen at ORCI during the period of 2006 through 2016. The study utilized the hospital registry of the ORCI hospital, and all the EC medical records of the hospital were abstracted for this study. Esophageal cancer cases were identified from the ORCI logbook as having been referred to or directly coming to ORCI with EC. The logbook contained each patient's name, medical record number, age, sex, and district of residence. ORCI routinely collects information on the permanent place of residence of the patient, in addition to the place of residence at the time of treatment. The address that was used in this study was the permanent place of residence. The medical record number from the logbook was then used to retrieve the corresponding medical record of each patient. Data for the period of 2006-2013 was obtained from our previous study dataset [7]. Logbook information from the paper medical records was used for data abstraction between 2006 and most of 2016 and combined with electronic medical records for the last four months of 2016. Variables abstracted from the paper and electronic medical record included tobacco use history, alcohol consumption history, tumor site, histopathological type of tumor, grade, stage, patient treatment (radiotherapy or chemotherapy), patient's religion, and referring hospital. Cases entered into the final dataset were verified by manually comparing them with the paper or electronic medical records. If a corresponding medical record could not be found due to occasional mislabeling in the logbook, data were considered missing and not included in the final analysis. Missing cases comprised 10.5% of all records. There were 1,332 cases identified as esophageal cancer between 2006 and 2013, plus 632 cases between 2014 and 2016, for 1,938 cases.

### 2.2. Risk Factors and Predictors

Possible risk factors for esophageal cancer were based on those from other settings similar to Tanzania [16, 18]. Those factors included the prevalences of smoking, poverty, unprotected water use, and solid fuel use [18]. Population-based data on smoking, unprotected water usage, and solid fuel use were obtained from the Tanzania 2015-2016 Demographic Health Survey (DHS) [19–21]. In addition, the population-based prevalence of poverty was obtained from the 2014 Tanzania United Nations Development Programme Income Report [22, 23]. Additional information from the Ministry of Health and Welfare in Tanzania was obtained regarding the number of healthcare facilities that were functioning during 2010-2016, including hospitals within each of the government Regions [23]. Then, the geodesic (~“straight line”) distance between the centroid of each Region and ORCI was calculated using the 2012 Regions shapefile “2012 PHC Shapefiles Tanzania Regional Profile” [24] from the Tanzania National Bureau of Statistics.

### 2.3. Statistical Analysis

Tanzania is administratively divided into 21 mainland Regions that have been grouped into 8 zones. Since Zanzibar has a separate government from the government of Tanzania and therefore is not part of the health care system of Tanzania or its governance, data from Zanzibar were not included in the analysis. Therefore, analyses were done for the residence of cases according to both regions and zones. In addition, regional annual incidence rates of EC from 2006 to 2016 were calculated for the 21 Tanzania mainland Regions using the ORCI data [22]. In addition, the annual incidence rates for EC were also calculated based on the Tanzania 2015-2016 DHS classification [22].

The overall average and yearly incidence rates of esophageal cancer per 100,000 persons were calculated at the regional level, as this provided greater statistical power with more data points. The yearly incidence rates were also calculated at the zone levels that might be more representative of true geographic variation. The population for each zone was based on the 2002 census projected population numbers [7]. Average regional and zonal incidence rates were calculated as the average annual number of cases per 100,000 persons treated at ORCI for that zone or region during the 2006-2016 period, divided by the average population of that zone or region between 2006 and 2016. Average annual incidence rates per 100,000 persons from 2006 to 2016 were calculated for these eight zones and compared yearly for zonal variation in EC. In addition, the annual incidence rate per 100,000 persons within each zone for 11 years between 2006 and 2016 was calculated with a chi-square test to evaluate temporal patterns of esophageal cancer in Tanzania.

Information about the number of hospitals in Tanzania was gathered for each of the 8 zones and 21 regions used in the analysis. ORCI collaborators and coauthors confirmed that esophageal cancer is clinically diagnosed at the regional hospital level in Tanzania. Poisson nonlinear regression was employed to examine the association between cases by zone compared to hospitals by zone and cases by region compared to hospitals by region. Poisson regression was chosen because the response and predictor variables were counted data from cancer cases at ORCI and hospital facilities across Tanzania. Results of this analysis were then plotted to examine the trend of the data with a coefficient of determination to approximate the model's goodness of fit. Results were further analyzed after removing Dar es Salaam because it is an extreme outlier. Finally, the Poisson regressions for cases by region compared to hospitals by region and cases by zone compared to hospitals were rerun without Dar es Salaam or Eastern Zone.

The next step was to evaluate the associations between cancer incidence rates and each of the four risk factors by region and zone. To develop zonal prevalence rates for these risk factors, the regional prevalences were multiplied by the estimated population for each region to create the estimated number of people in each region affected by each etiologic factor. Next, the number of people affected by a particular etiologic factor from each region in the same zone was summed to obtain a zonal number of people affected by that etiologic factor. This numerator was divided by the zonal population to form a new prevalence value for that etiologic factor by zone. Poisson regression of the zonal and regional incidence rates compared to these four etiologic factors was also used to determine if there was a nonlinear trend due to cancer count data being count data and the predictor variable being continuous data. The natural log for these Poisson regressions was then taken to interpret the results more easily. No confounders were evaluated in our study.

Data were analyzed using Microsoft Excel, SAS 9.4 Software, R Studio, and ArcMap 10.5.1. The study was approved by the Institutional Review Boards (IRBs) of the University of Michigan and ORCI in Tanzania.

## 3. Results

A total of 1,938 esophageal cancer patients were identified and included in this study (Table 1). Summary results indicated that patients tended to be older and male, with a large percentage using alcohol and tobacco (Table 2). The average age of patients was 59 years, 68% of whom were male and 32% were female. Overall, 64% of patients either smoked tobacco, consumed alcohol, or both, compared to 34% that did neither. The histopathological type of esophageal cancer was largely squamous cell carcinoma at over 90% compared to less than 10% presented with adenocarcinoma. There was a general increase in cases per year (Table 1), but this varied, with some years, having fewer cases than previous years (for example, years 2011-2013).

Average incidence per 100,000 individuals, cases per hospital, etiologic risk factors, and distance from the patient's residence to ORCI varied by Zones and Regions (Table 3). In general, the Dar es Salaam Region and the Eastern Zone showed the highest incidence rate, cases per hospital, and defined etiologic risk factors. Dar es Salaam and the Eastern Zone also had the lowest prevalence of poverty, unprotected water use, and solid fuel source use. These observations were not unexpected, as Dar es Salaam is the largest city in Tanzania, sharing some of the country's administrative functions with Dodoma. Dar es Salaam also has many health facilities, including ORCI, where cancer diagnosis and treatment are available. The Lake Region had a very low incidence of EC referrals to ORCI, averaging 15 times less than the Eastern Region. Table 3 illustrates the geographic breakdown of the incidence rates, the average number of persons per hospital, and the prevalence of risk factors.

During each of the 11 years from 2006-2016, there was a statistically significant difference among zones in the yearly incidence rates per 100,000 (Table 4). The incidence per 100,000 from 2006 to 2016 was compared within each zone to the average incidence of that zone to evaluate whether there were zone-specific differences per year. None of the p-values for any of the Zones were statistically significant.

The association between Zonal and Regional cancer incidence per 100,000 and the number of hospitals was positively significant when all cases were analyzed (Table 5). Among zones, each additional hospital was associated with an increase in the incidence of esophageal cancer by 1.17 times the previous incidence (95% CI 1.04, 1.32; *P* value < 0.01) for the Zonal Poisson analysis. For the regions, each additional hospital was associated with an increase in the incidence of EC by 1.60 times (95% CI 1.36, 1.88; *P* value < 0.01) for the Regional Poisson analysis.

Poverty was the only significant risk factor associated with EC incidence (Table 6). For the Regional Poisson analysis, the parameter estimates of 0.66 indicate that with greater poverty, the risk of cancer decreased (0.66, 95% CI 0.54, 0.79; *P* value < 0.01), given that the other covariates are held constant. Among the zones, a one percent increase in poverty prevalence was associated with a decrease in EC incidence by 0.57 times that of the previous incidence rate (95% CI 0.41, 0.78; *P* value < 0.01) for the Zonal Poisson analysis. However, in multivariate Poisson regression analysis, when distance and number of hospitals were added as predictors to the Region, the distance was the only statistically significant predictor and was inversely associated with regional esophageal cancer (Table 7). In addition, individually adding hospitals and distance to the four etiologic risk factors resulted in a greater than 10% change in the parameter estimates for the regional tobacco, unprotected water, solid fuel source, and prevalence of poverty covariates.

**Figure 1:**
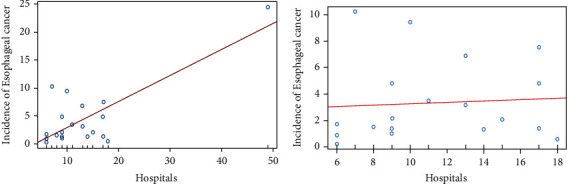
Associations between the number of hospitals and esophageal cancer incidence with or without Dar es Salam hospital.

Excluding Dar es Salaam from the Regional incidence rate and hospital analysis, as well as excluding the Eastern Zone from the zonal incidence rate and hospital analysis, removed this significant relationship between EC incidence and hospitals. A positive linear trend was observed when the association between regional incidence rates and hospitals was plotted (Figure 1(a)). However, when the outlier value of Dar es Salaam was removed, this trend was weakened to almost no observable association between the regional number of hospitals and EC incidence (Figure 1(b)). In addition, we calculated associations by Poisson regression with Dar es Salaam removed for the Regional analysis and the Eastern Zone removed for the zonal analysis (Table 5). With the influential observation removed, the association between incidence and the number of regional or zonal hospitals was no longer statistically significant.

## 4. Discussion

The primary goal of this study was to investigate population-level geographic patterns in esophageal cancer cases that received treatment at Ocean Road Cancer Institute (ORCI) in relation to local risk factors. The study revealed statistically significant differences in the incidence of EC among geographical zones. The study also illustrated that poverty was negatively associated with the incidence of EC at the levels of Region and Zone, which may reflect underdiagnoses, lack of ability of transportation to advanced medical centers, or decreased access to care. In addition, important regional risk factors were identified that could be difficult to access in case-control studies and should be considered in future studies. This analysis reaffirmed the previous findings by Gabel and colleagues [7], who also observed regional differences during 2006-2013. In addition, temporal variation within zones was evaluated to determine if EC epidemiology had changed during the eleven-year study period. The analysis of within-zone annual variation suggests a roughly constant rate of EC during 2006-2016. In general, zones and regions with more dense, urbanized populations were closer to ORCI and had higher incidence rates. This result is illustrated by comparing, for example, Dar es Salaam in the Eastern Zone with zones in more remote and rural areas such as Shinyanga in the Lake Zone. More urbanized regions also tended to have hospitals with smaller population catchments than more rural regions. In addition, our results showed that areas with lower incidence rates of EC tended to be farther away from ORCI.

Other studies have also investigated esophageal cancer in relation to lifestyle risk factors, access to diagnosis at hospitals, and distance to treatment. In Kenya, low socioeconomic status, smoking, snuff use, alcohol, tooth loss, cooking with charcoal and firewood, hot beverage use, and use of mursik were independently associated with EC [16]. However, the results from our study in Tanzania differ in that none of the four population-level risk factors that we were able to examine (tobacco use prevalence, unprotected water use prevalence, solid fuel source prevalence, and poverty prevalence) were significantly associated with EC in the final model. A possible explanation for this difference is that the Kenyan study was a case-control study with risk factor information for both cases and controls, compared to our cross-sectional study, which looked at the population prevalence of risk factors by region and not for individual cases. Other studies have shown that smoking and alcohol consumption are risk factors for esophageal cancer cases. [25, 26]. Risk factors on an individual case level are still likely the primary drivers of developing EC in this setting, even if they do not explain regional variation in incidence.

Our study has shown that access to care barriers such as distance to treatment may be more important than population-level risk factors in understanding geographic differences in EC in Tanzania. Therefore, early EC cases could be missed for various reasons. Diagnosis of EC is difficult as systems could be nonspecific Gastrointestinal reflux Disease- (GERD-) like symptoms [27]. First, remote areas of Tanzania may not be served by a regional hospital that can provide diagnostic laboratory services and radiology, compared to ORCI, which provides these services and can aid in detecting EC [28–30]. Second, many remote areas lack the endoscopic equipment and endoscopists that would allow for greater detection of EC independent of laboratory and radiology services [30]. Increasing the capacity of referral hospitals to offer more advanced diagnostic laboratory and radiology services and providing greater endoscopic equipment and training would allow for earlier detection of EC., Patients with esophageal cancer in remote areas face more travel barriers (transportation costs, time) to receive a diagnosis at ORCI. Therefore, the low EC prevalence may be due to underdiagnosis.

Furthermore, poverty, distance to the ORCI, and inadequate access to health care likely contributed to late diagnosis, misdiagnosis, and underrepresentation of EC in remote areas of Tanzania. In addition, improving the ability of patients in more remote regions to travel to seek care should help to reduce underreporting of EC. Finally, there is a general shortage of equipment capable of diagnosis of EC as well as insufficient facilities in the region to tackle this problem. Thus, this study implicitly raises awareness of how improving access to health care at the ORCI cancer center should enhance accurate diagnosis and treatment success.

Since ORCI is the main facility in Tanzania currently performing chemotherapy and radiotherapy, improving access to this facility by reducing the burden of travel on remote populations will be important in creating equitable esophageal cancer care for Tanzanians. Thus, the population of esophageal cancer cases at ORCI will likely underestimate the true extent of EC across Tanzania. Second, there may be considerable underreporting of cases in certain areas and among some populations if patients lack the financial resources or physical capacity to travel to ORCI for treatment. Third, a small number of EC cases were being treated with chemotherapy at Kilimanjaro Christian Medical Centre and Bugando Medical Centre from 2013-2016, possibly adding to the underreporting of cases near these hospitals.

Our study has some limitations. First, the data from ORCI are from hospital-based registries, and there is no defined catchment area for cases. Therefore, some EC cases in this dataset were referred from other Regions. Second, the notion that esophageal cancer presents with nonspecific symptoms at first makes it harder to diagnose the disease at its early stages. Third, for the Poisson regressions, regional analysis had to be restricted to 21 mainland regions instead of the 25 current Regions due to the formation of 4 additional regions that were recently created but did not have stratified data before 2012. As a result, the geodesic distance underestimates the actual distance and may not reflect the relative time of travel that is related to vehicle availability and accessibility of roads. Also, as is done for most studies like ours, Euclidian or “crow fly” distance between the regional center and ORCI was used as a proxy measure. Thus, ORCI to Dodoma was measured as roughly 385 kilometers. This Euclidian distance represents a relative distance comparison among regions that represents how relatively difficult ORCI is to access. We recognize that there are limitations to this, as travel will be more or less difficult depending on the topography and infrastructure of each region. Finally, the multivariable Poisson regression was done as a regional analysis because there was no appropriate Zonal shapefile to analyze those finer-scale distances.

Among the strengths of this study is the large number of carefully diagnosed, well-documented EC cases that were analyzed, spanning 11 years. These data provide a valuable resource to build upon, given that data were collected in a low-resource setting. Indeed, there is very limited information about esophageal cancer risk in Eastern Africa [31, 32]. Smoking and heavy drinking have been suggested to be significant risk factors for esophageal squamous carcinoma and adenocarcinoma [29, 33, 34] and should be more carefully examined through a clinic-based study. While multiple risk factors have been identified as associated with esophageal cancer: socioeconomic status, malnutrition, smoking, alcohol use [7], and diet tend to be the main risk factors linked with greater esophageal cancer risk [2, 13–17]. Malnutrition is common in patients with EC as the esophagus is the gateway to the gastrointestinal tract [18].

Further, cachexia in patients with EC contributes to the added risk of malnutrition and the need for nutrition support for patients with EC [19, 20]. Although nutrition and dietary factors have been suspected in the etiology of esophageal cancer, these relationships need further study. A National Nutrition Institute could be involved in future studies on EC in Tanzania. Dietary assessment tools such as food frequency questionnaire, hot food and beverages intake, and nutrient intake would be informative. It is noteworthy that the age-standardized cancer rate is 8.9 per 100,000; that rate in men (11.7) is 1.75 times greater than that in women (6.7 per 100,000) [35]. This ORCI esophageal cancer database may serve as the basis for such studies in Tanzania.

Our ability to estimate population-level data on the distance to and the number of hospital facilities using the Tanzanian Health Facility Registry could allow for further analysis of differences in access to treatment and diagnosis. The inclusion of distance from ORCI to the centroid of the regions as a predictor of access to care can help explain differential underreporting of EC. Efforts to improve early diagnosis of EC include gastric endoscopy and improving access to diagnostic and treatment facilities by reducing the travel burden to remote populations. Although this study analyzed certain population-level risk factors stratified by region from the 2015-2016 DHS and the 2014 Tanzania United Nations Development Programme Income Report, future studies should further address the prevalence of individual risk factors for the development of esophageal cancer in Tanzania. Additionally, a longitudinal study may answer several interesting questions that could not be addressed in this retrospective study; therefore, future longitudinal studies when resources are available would be valuable.

## Figures and Tables

**Table 1:** Distribution and number of esophageal cancer cases from Ocean Road Cancer Institute (ORCI) from the year 2006-2016.

Total ORCI cases per year (*n* = 1938)	*N* (percent)
2006	131 (6.8)
2007	135 (7.0)
2008	163 (8.4)
2009	195 (10.1)
2010	204 (10.5)
2011	158 (8.2)
2012	122 (6.3)
2013	141 (7.3)
2014	231 (11.9)
2015	236 (12.2)
2016	222 (11.5)
Total confirmed cases	1938
Missing cases	227 (10.5%)

**Table 2:** Demographics, risk factor, and histopathology of esophageal cancer cases from Ocean Road Cancer Institute.

Demographic characteristics	Mean
Average age	58.54
Sex	*N* (percent)
Male	1301 (68.04)
Female	611 (31.96)
Modifiable behaviors	
Tobacco usage only	160 (9.99)
Alcohol usage only	219 (13.67)
Alcohol and tobacco usage	639 (39.89)
Neither tobacco or alcohol usage	584 (36.45)
Histopathology	
Squamous cell carcinoma	1146 (90.67)
Adenocarcinoma	118 (9.34)

**Table 3:** Zonal and regional average incidence rate (IR^∗^) from 2006-2016, access to diagnosis, and population-level risk factors.

Zone	Average IR	Persons per hospital	Tobacco use (%)	Unprotected water (%)	Solid fuel source (%)	Incidence of poverty (%)	Distance from ORCI (km)
Region							
Central	3.05	185,767	12.5	47.4	96.6	61.9	
Dodoma	4.80	238,601	11.4	40.5	97.3	54.3	385
Manyara	1.39	160,384	14.1	45.0	96.3	65.8	397
Singida	2.15	155,191	13.1	60.4	95.9	69.3	543
Eastern	15.94	91,627	14.3	26.7	88.1	43.7	
Dar es Salaam	24.46	62,990	13.6	13.0	80.2	25.8	^∗∗^
Morogoro	6.87	165,613	19.5	36.2	95.9	62.5	278
Pwani	10.25	154,679	11.4	48.0	96.1	59.2	81
Lake	0.71	219,370	9.6	50.2	97.2	72.4	
Kagera	1.35	190,144	7.8	62.5	98.2	72.2	1,045
Mara	1.00	210,155	9.3	44.1	96.9	74.8	830
Mwanza	0.58	202,778	14.8	42.3	96.7	68.9	782
Shinyanga	0.25	666,472	9.6	51.8	97.2	74.5	
Northern	6.83	134,740	17.7	29.6	87.9	53.6	
Arusha	3.17	130,845	11.4	19.5	76.5	55.0	570
Kilimanjaro	7.52	97,731	23.4	13.6	89.6	33.5	388
Tanga	9.44	200,246	18.8	51.5	96.3	68.8	213
Southern	1.51	158,651	22.9	64.1	97.7	74.4	
Lindi	1.38	104,303	19.5	65.4	97.6	81.9	305
Mtwara	1.71	223,932	24.8	63.3	97.8	69.2	432
Southern highlands	4.51	113,336	13.4	40.9	97.6	61.7	
Iringa	4.79	103,061	2.3	42.6	97.3	60.5	432
Ruvuma	3.48	128,047	23.7	38.9	97.9	63.1	545
South west highlands	1.90	133,882	15.1	49.1	97.0	66.4	
Mbeya	2.09	181,799	12.9	44.1	96.6	63.3	669
Rukwa	1.41	91,602	16.6	57.7	97.6	71.6	887
Western	1.26	311,713	12.3	56.4	98.2	74.8	
Kigoma	0.90	315,313	15.6	38.5	98.2	72.8	1,008
Tabora	1.56	305,024	10.3	70.4	98.2	76.4	736

^∗^Incidence rate (IR) is calculated as cases per 100,000 persons.

**Table 4:** Yearly incidence rate (IR^∗^) variation between zones from 2006-2016.

Year	Central IR	Eastern IR	Lake IR	Northern IR	Southern IR	Southern highlands IR	Southwest highlands IR	Western IR	Average yearly IR	*P* value^∗∗^
2006	2.08	15.91	0.49	3.35	1.93	3.51	1.10	1.70	3.76	<0.01
2007	3.81	11.46	0.57	4.70	0.95	3.77	1.33	1.08	3.46	<0.01
2008	2.18	15.34	1.09	4.58	1.39	3.02	1.54	2.08	3.90	<0.01
2009	3.60	16.59	0.88	6.42	0.91	4.27	3.22	1.25	4.64	<0.01
2010	2.27	16.68	0.59	7.97	1.34	4.82	2.88	1.68	4.83	<0.01
2011	2.20	13.98	0.49	5.38	0.44	4.09	0.93	0.92	3.55	<0.01
2012	1.55	10.01	0.47	4.53	0.43	2.47	1.35	0.44	2.66	<0.01
2013	3.02	9.06	0.53	5.68	2.10	3.33	1.53	0.43	3.21	<0.01
2014	2.94	17.88	0.74	6.95	2.06	6.24	1.27	2.05	5.02	<0.01
2015	3.58	17.52	0.71	7.99	2.03	5.26	1.85	0.79	4.97	<0.01
2016	2.79	15.08	0.55	9.66	1.43	4.02	2.00	0.57	4.51	<0.01
Average	2.77	14.49	0.64	6.21	1.38	4.10	1.73	1.15	4.06	<0.01
*P* value^∗∗∗^	0.99	0.79	1.00	0.84	0.99	0.99	0.98	0.97	1.00	

^∗^Incidence rate (IR) is calculated as the number of cases per 1,000,000 persons in each zone. ^∗∗^Zonal variation in esophageal cancer by year was statistically significant using a chi-square test for independence at a *P* value of 0.05 for every Zone. ^∗∗∗^Annual variation in esophageal cancer by zone was not statistically significant at a *P* value ≤ 0.05.

**Table 5:** Poisson regressions for the association between regional hospitals for diagnosis of esophageal cancer and incidence rate (IR) of esophageal cancer (EC).

Poisson regressions stratified by geography compared to incidence rate	Degrees of freedom	Effect estimate	95% confidence limits	*P* value^∗^
Association between zonal hospitals and EC IR^∗^	6	1.17	(1.04, 1.32)	<0.01
Association between regional hospitals and EC IR^∗∗^	19	1.60	(1.36, 1.88)	<0.01
Association between zonal hospitals and EC IR without eastern zone	5	1.02	(0.92, 1.13)	0.74
Association between regional hospitals and EC IR without Dar es Salaam	18	1.06	(0.76, 1.46)	0.73

^∗^ *P* value is significant at 0.05.

**Table 6:** Poisson regressions for the association between prevalence of etiologic risk factors for esophageal cancer and incidence rate.

Risk factor prevalence and incidence rate multiple linear regressions	Degrees of freedom	Parameter estimate	95% confidence limit	*P* value^∗^
Regional risk factors/incidence rate	16			
Tobacco use		0.99	(0.78, 1.27)	0.95
Unprotected water use		1.15	(0.97, 1.36)	0.10
Solid fuel source use		0.87	(0.61, 1.23)	0.44
Incidence of poverty		0.66	(0.54, 0.79)	<0.01
Zonal risk factors/incidence rate	3			
Tobacco use		0.84	(0.58, 1.22)	0.38
Unprotected water use		1.32	(0.94, 1.86)	0.10
Solid fuel source use		0.68	(0.36, 1.26)	0.22
Poverty		0.57	(0.41, 0.78)	<0.01

^∗^ *P* value is significant at 0.05.

**Table 7:** Poisson regression for the association between prevalence of etiologic risk factors for esophageal cancer and distance with incidence rate.

Risk factor prevalence and incidence rate multiple linear regressions	Degrees of freedom	Parameter estimate	Wald 95% confidence limit	Wald-chi-square	*P*-value^∗^
Regional risk factors/incidence rate	13				
Tobacco use		-0.0436	(-0.1774, 0.0903)	0.53	0.5236
Unprotected water use		-0.0102	(-0.1086, 0.0882)	0.41	0.8385
Solid fuel source use		0.0862	(-0.1116, 0.2840)	0.04	0.3932
Poverty		-0.1029	(-0.2414, 0.0356)	0.73	0.1452
Hospitals		0.0321	(-0.1825, 0.2468)	2.12	0.7692
Distance (km)		-0.0074	(-0.0108, -0.0040)	0.09	<0.0001

^∗^ *P* value is significant at 0.05.

## Data Availability

Access to data will be available upon written request to the corresponding author.
